# Supplementary figures and images for: Peripheral circadian rhythms in the liver and white adipose tissue of mice are attenuated by constant light and restored by time-restricted feeding
Source: PLoS One. 2020 Jun 12;15(6):e0234439. doi: 10.1371/journal.pone.0234439 (PMC7292356; doi:10.1371/journal.pone.0234439)

S1 Fig

A

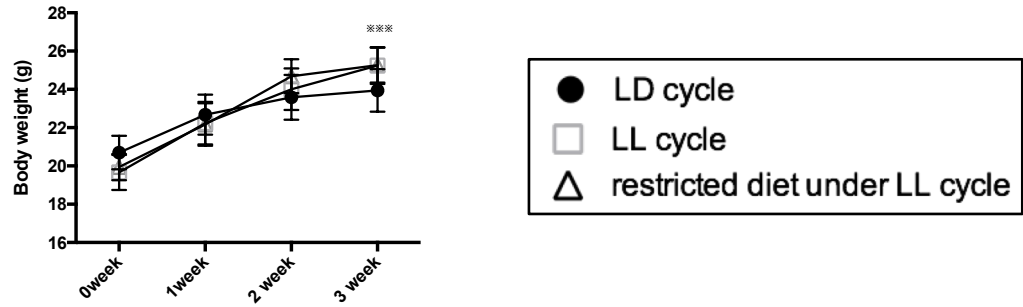

B

Liver

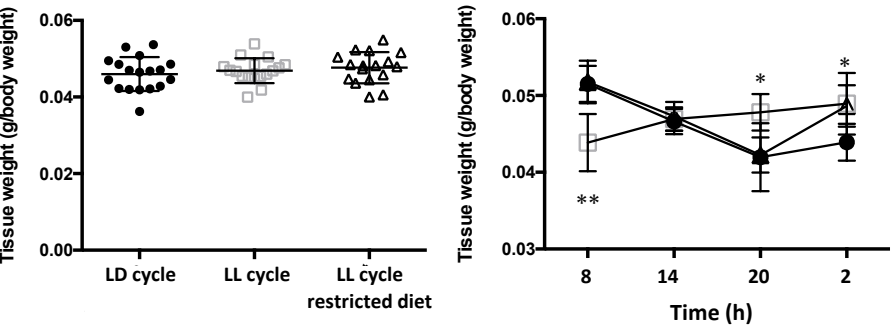

C

WAT

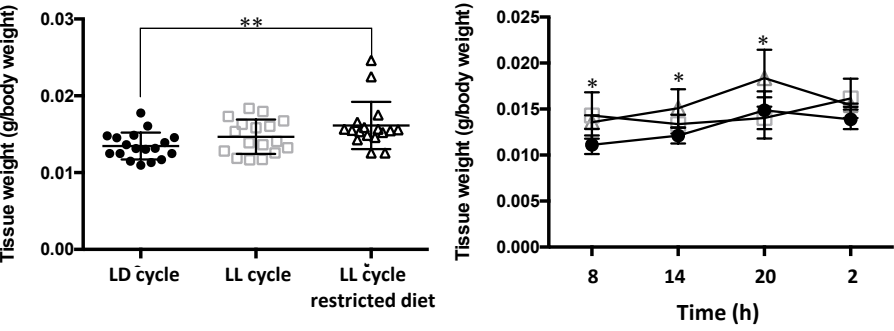

D

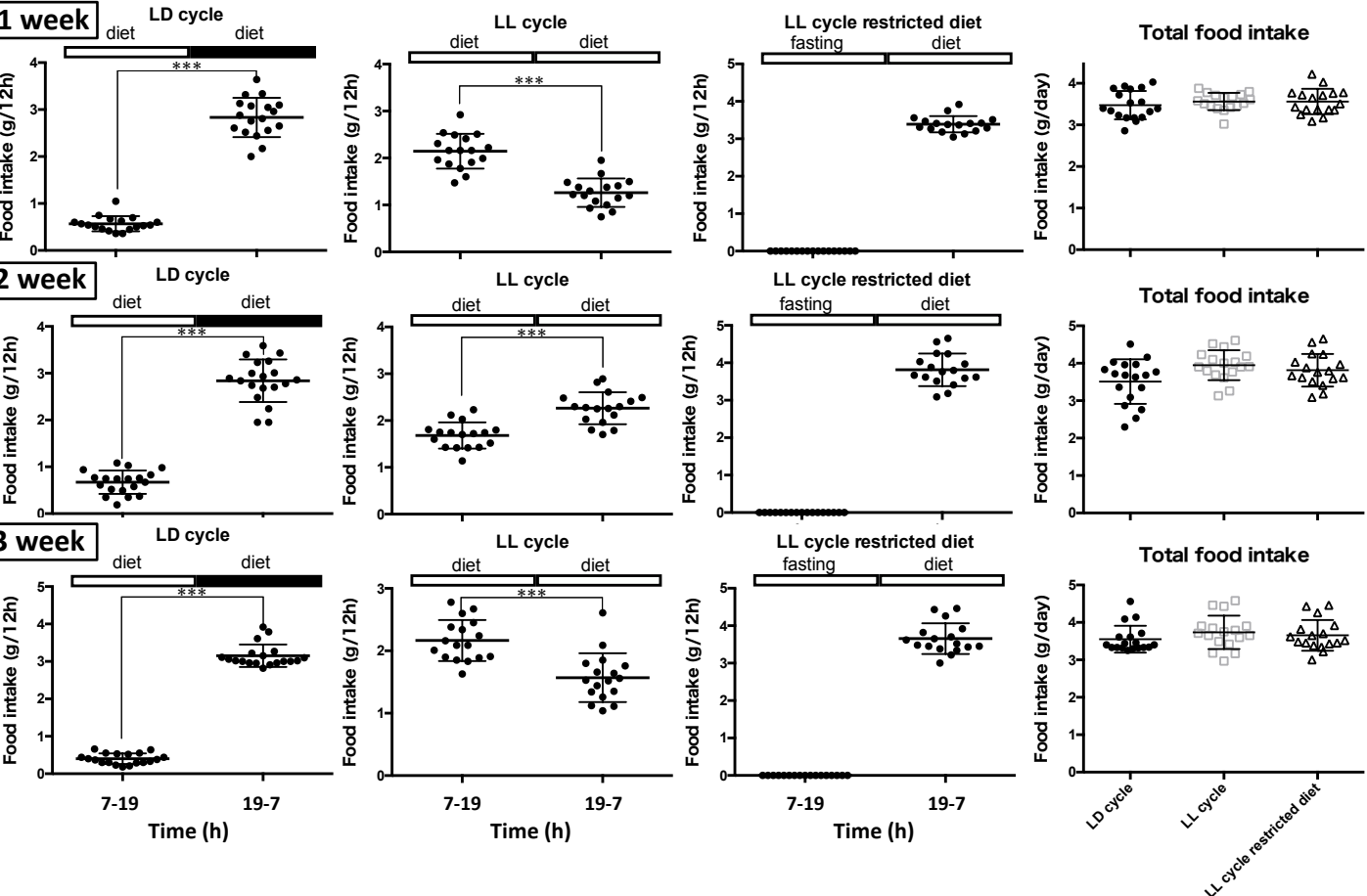

Supplement: S1 Fig — (A) Changes in body weight (each group, n = 16). (B) liver weight and (C) WAT weight were measured at 3 weeks under different housing conditions (closed circle, ad libitum under LD cycle; open square, ad libitum under LL cycle; open triangle, restricted feeding at night-time under LL cycle). (D) Food intake was measured at 07:00–19:00 and 19:00–07:00 under different housing conditions. Values indicate the mean ± SD. * p < 0.05, ** p < 0.01, *** p < 0.001, LD cycle vs LL cycle, LD cycle vs restricted feeding under LL cycle determined by two-way ANOVA and one-way ANOVA test for A-C, and by paired Student’s t tests and one-way ANOVA test for D. (PDF) [file pone.0234439.s001.pdf]

S2 Fig

Liver

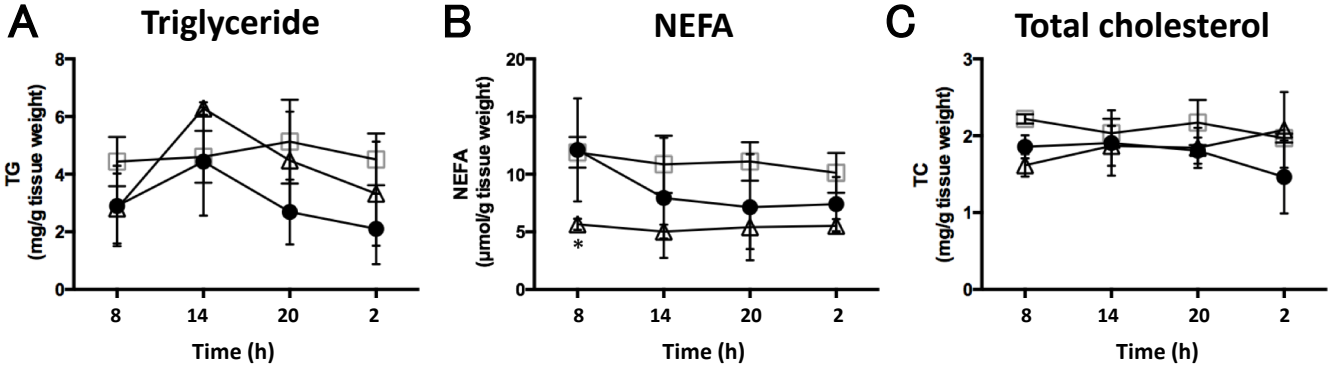

WAT

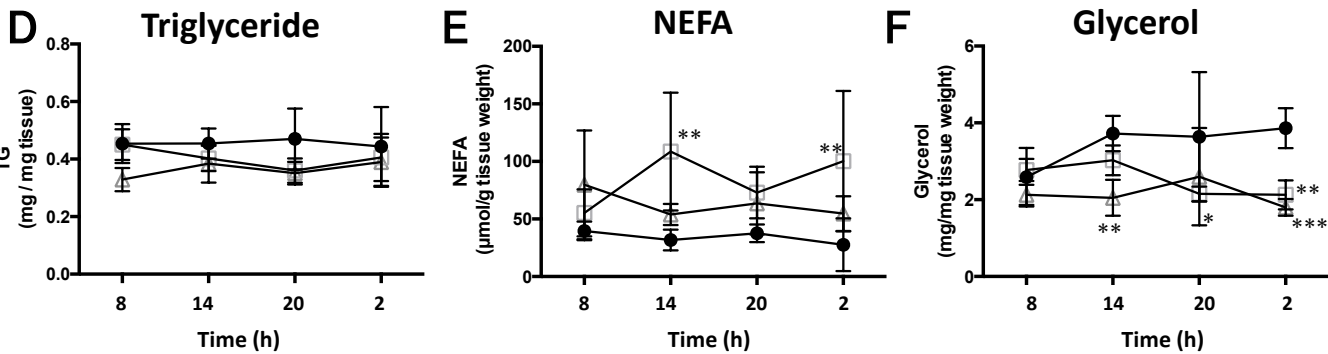

plasma

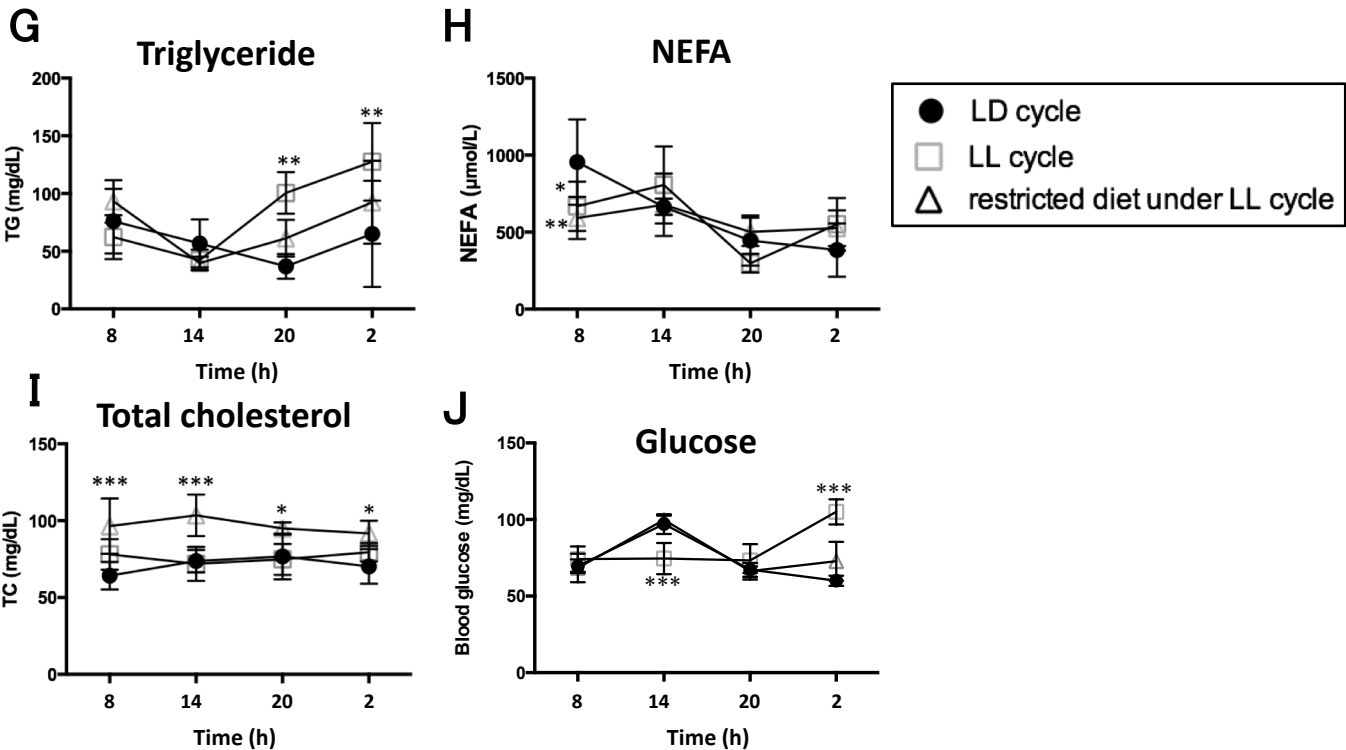

Supplement: S2 Fig — (A) Triglyceride, (B) non-esterified fatty acids and (C) total cholesterol were measured in the liver housed under different conditions (closed circle, ad libitum under LD cycle; open square, ad libitum under LL cycle; open triangle, restricted feeding at night-time under LL cycle) at each time points (08:00, 14:00, 20:00 and 02:00) using enzymatic colorimetric assay kits. Plasma was collected at each time points (08:00, 14:00, 20:00 and 02:00). (D) Triglyceride, (E) non-esterified fatty acids and (F) glycerol in WAT were measured by enzymatic colorimetric assay kits. Plasma (G) triglyceride, (H) non-esterified fatty acids, (I) total cholesterol and (J) glucose were measured. Values indicate the mean ± SD of n = 4 mice per group for each time point. * p < 0.05, ** p < 0.01, *** p < 0.001, LD cycle vs LL cycle, LD cycle vs restricted feeding under LL cycle determined by two-way ANOVA followed by the Dunnett’s multiple comparisons test. (PDF) [file pone.0234439.s002.pdf]
